# An ultra high-endurance memristor using back-end-of-line amorphous SiC

**DOI:** 10.1038/s41598-024-64499-2

**Published:** 2024-06-18

**Authors:** Omesh Kapur, Dongkai Guo, Jamie Reynolds, Daniel Newbrook, Yisong Han, Richard Beanland, Liudi Jiang, C. H. Kees de Groot, Ruomeng Huang

**Affiliations:** 1 School of Electronics and Computer Science, University of Southampton, Southampton, SO17 1BJ UK; 2 Department of Physics, University of Warwick, Coventry, CV4 7AL UK; 3 School of Engineering, University of Southampton, Southampton, SO17 1BJ UK

**Keywords:** Memristor, SiC, Back-end-of-line, PECVD, Endurance, Electronic devices, Electronic devices

## Abstract

Integrating resistive memory or neuromorphic memristors into mainstream silicon technology can be substantially facilitated if the memories are built in the back-end-of-line (BEOL) and stacked directly above the logic circuitries. Here we report a promising memristor employing a plasma-enhanced chemical vapour deposition (PECVD) bilayer of amorphous SiC/Si as device layer and Cu as an active electrode. Its endurance exceeds one billion cycles with an ON/OFF ratio of *ca.* two orders of magnitude. Resistance drift is observed in the first 200 million cycles, after which the devices settle with a coefficient of variation of *ca.* 10% for both the low and high resistance states. Ohmic conduction in the low resistance state is attributed to the formation of Cu conductive filaments inside the bilayer structure, where the nanoscale grain boundaries in the Si layer provide the pre-defined pathway for Cu ion migration. Rupture of the conductive filament leads to current conduction dominated by reverse bias Schottky emission. Multistate switching is achieved by precisely controlling the pulse conditions for potential neuromorphic computing applications. The PECVD deposition method employed here has been frequently used to deposit typical BEOL SiOC low-k interlayer dielectrics. This makes it a unique memristor system with great potential for integration.

## Introduction

The ever-increasing complexity and data intensity of modern technological applications has been the driving force for the development of novel non-volatile memory solutions that enable fast and power-efficient computation^1,2^. Resistive Random-Access Memory (RRAM) have attracted great research interest for next-generation non-volatile memory because of their attractive switching performance and scaling potential^3^. RRAM have a metal–insulator-metal structure where an insulating layer is sandwiched between two electrodes^4^. The formation and disruption of the nanometre scale filament(s) in the insulating layer due to the ion migration in the presence of an electric field leads to the low resistance state (LRS) and high resistance state (HRS), respectively^5^. The potential to control the filament size and hence resistance state in a near analogue way gives rise to the potential to emulate the behaviour of synapses and neurons for neuromorphic computing^6–8^.

Both cation and anion can dominate the ion migration in the RRAM technology^8^. Anion-based RRAM is normally referred to as Valence Change Memory (VCM). It involves the migration of anions (e.g. O, S) within the insulating layer to create a conductive pathway^9^. On the other hand, cation migration uses donated ions from the top electrode to create the conductive pathway and is referred as Electrochemical Metallization Memory (ECM). This donation requires using an active electrode such as Cu or Ag^10^. Both ECM and VCM enjoy strengths such as fast switching, multi-level states and excellent retention^11^. Compared with VCM, ECM devices have higher ON/OFF ratio and can operate at much lower current and voltages^12,13^. However, VCM is still considered the preferred switching mode given its superior performance especially in its switching endurance^14^. VCM normally registers a much higher endurance over ECM, with the largest respective endurances reported as 10^12^ and 10^8^ switching cycles^15,16^. The large variation in endurance is thought to be due to the differences in the migration type^17^. VCM utilises a native dielectric species, while ECM suffers from a filament formed through foreign ionic species from the active electrode. This could cause stress to the dielectric film and lead to permanent plastic deformation^18^. As well as this the filament shape and contact point may not be consistent over the switching cycle. Development of an ECM device that enables large endurance can therefore be extremely beneficial to leverage its advantages of high ON/OFF ratio and low operation currents.

Apart from high switching performance, the capability of integrating ECM cells directly into a CMOS low-k/Cu interconnect lines would be particularly advantageous, as it could help reduce latency in connectivity-constrained computational devices and reduce a chip’s footprint by stacking memory directly on top of logic. This reduces the distance between the standard CMOS memory and ECM in-memory computing modules, creating a faster overall performance. The potential functionality of these integrated devices has led to significant effort toward making resistive memory compatible with BEOL integration^19,20^. Common native low-k dielectric BEOL materials include amorphous Si, SiO_2_, SiN, SiC or combinations of these materials. SiO_2_ could be considered the archetype solid electrolyte resistive memory material and has been extensively investigated for many years using sputtered films or layers formed by low-pressure chemical vapour deposition (LPCVD)^21,22^. Charge trapping-induced resistive-switching was also reported in sputtered SiON^23^. The chemical vapour deposition route is strongly preferred as it is compatible with mainstream BEOL fabrication processes and thus could be potentially embedded in BEOL, addressing interconnect latency issues^24^. SiCN resistive memory by plasma-enhanced atomic layer deposition and plasma-enhanced chemical vapour deposition (PECVD) has led to memory with promising endurance (10^6^ cycles) with recent improvements in switching characteristics by added interfacial layers^25,26^. Amorphous Si by PECVD shows basic resistive switching functionality^27,28^. Fully native PECVD SiOC: H has shown high ON/OFF ratios of more than six orders of magnitude, though with limited endurance^13,29^.

Among all BEOL materials, amorphous silicon carbide (SiC) is regarded as the third-generation semiconductors due to its superior electrical properties such as large band gap, high breakdown voltage, good thermal conductivity, and has received significant research attention^30,31^. Amorphous SiC based resistive memory using sputtered SiC as the insulating layer together with a Cu active electrode was first reported by Lee and colleagues, who demonstrated a 10^5^ endurance with an ON/OFF ratio of two orders of magnitude and low power consumption with an added thermal GeSbTe barrier^32,33^. Our research using sputtered SiC as a memory device layer has resulted in devices with a record nine orders of magnitude ON/OFF ratio though low endurance (< 50 cycles)^34,35^. Promising radiation hardness against γ-rays up to 2 MRad was also reported for SiC memoristors^36^. Recently, we have successfully fabricated PECVD amorphous Si-rich SiC resistive memory. The Si-rich SiC has a significant amount of Si–Si bonds, which could facilitate the migration of Cu ions in the device layer. These memristors show excellent switching without a requirement for electroforming with up to 10^3^ ON/OFF ratio^26^. Unlike sputtered amorphous SiC, the PECVD amorphous SiC memories also demonstrate short-term plasticity when stimulated with a series of designed electrical pulses, leading to the demonstration of synaptic functions^26^. Such volatile switching behaviour was exploited to serve as the physical reservoir that could enable temporal signal processing^37^. Despite the previous progress, SiC based memristor still suffers poor switching stability due to the random growth of Cu filaments in the SiC layer which leads to limited endurance. It was reported that engineering of the material stack in a memristor can potentially help control filament growth and improve its performance. One notable example is the adoption of bilayer structure in the memristor which can improve the memory stability primarily through confining the conductive filament in the stable layer^38,39^.

Here, we report the growth and device performance of a PECVD SiC/Si bi-layer resistive memory. We demonstrate that the SiC/Si memory cells show well-controlled bipolar and unipolar switching behaviour. The resistance states can be precisely controlled by varying the pulse conditions to show multistate switching. More importantly, our bilayer SiC/Si memristors demonstrate an endurance of more than 1 billion cycles at an ON/OFF ratio of *ca.* 10^2^. The combined advantages of using the PECVD method and extremely high endurance performance promises this to be a new class of resistive memory devices with great potential for implementation.

## Results and discussion

**Figure 1:**
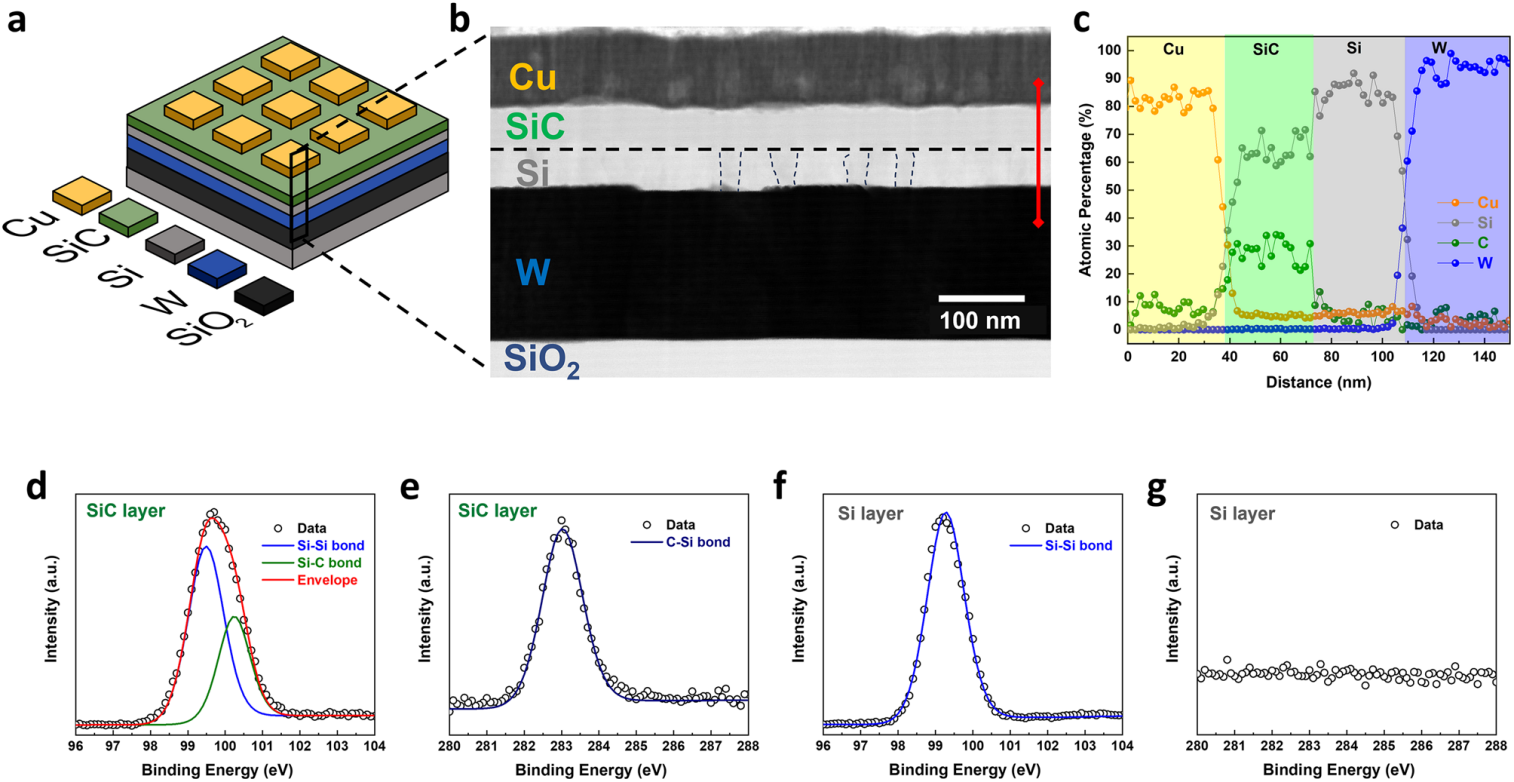
Characterization of the Cu/SiC/Si/W bilayer memristor. (**a**) Schematic of the Cu/SiC/Si/W memristor structure. (**b**) Cross-sectional TEM image and (**c**) the associated EDX analysis of the fabricated memristor. XPS core-level spectra of (**d**) Si 2*p* and (**e**) C 1*s* within the SiC layer, and XPS core-level spectra of (**f**) Si 2*p* and (**g**) C 1*s* within the Si layer.

Figure 1a presents the schematic of the bilayer SiC memristor fabricated in this work. Transmission electron microscopy (TEM) cross-sectional image was taken from the memristor, as shown in Fig. 1b. The distinctive layers of the Cu and W electrodes are clearly visible at the top and bottom where the Si/SiC bilayer is sandwiched in between. It can be observed that the SiC layer is characterised with an amorphous texture. On the other hand, several grey patterns can be spotted in the underneath Si layer, suggesting the formation of nanoscale grain boundaries (highlighted with the dotted lines). The polycrystalline nature of the Si layer was confirmed with X-ray diffraction (XRD) and is shown in Fig. S1. The composition of these two layers were investigated by conducting an Energy-dispersive X-ray spectroscopy (EDX) scan across the cross-sectional region (scanning range marked by the red line in Fig. 1b) as shown in Fig. 1c. The SiC layer was found to be Si rich with a Si to C atomic ratio of *ca.* 7:3. The Si layer underneath shows no sign of C, indicating the existence of a separate, pure Si layer. X-ray photoelectron spectroscopy (XPS) depth-profile measurement directly on the SiC/Si film further confirmed the bilayer structure where the measured Si and C atomic concentrations align well with the EDX results (Fig. S2a). Figure 1d–g plot the core-level XPS spectra of Si 2*p* and C 1*s* to analyse the chemical structure of these two layers (respective survey spectra are shown in Fig. S2b). Figure 1d shows the Si 2*p* spectrum in the SiC layer can be deconvoluted into two sub-peaks at 99.4 eV and 100.2 eV, attributable to the metallic Si^0^ (Si–Si bonding) and the Si–C bonding, respectively^40,41^. The C 1*s* spectrum in the SiC layer is presented in Fig. 1e, showing a single peak at 283.0 eV which represents the C–Si bonding. On the other hand, the Si 2*p* spectrum in the Si layer is characterised by one single peak at 99.4 eV, indicating that only Si–Si bonding is available in the layer (shown in Fig. 1f). This is further validated by the absence of a C peak in its C 1*s* spectrum scan in Fig. 1g.

**Figure 2:**
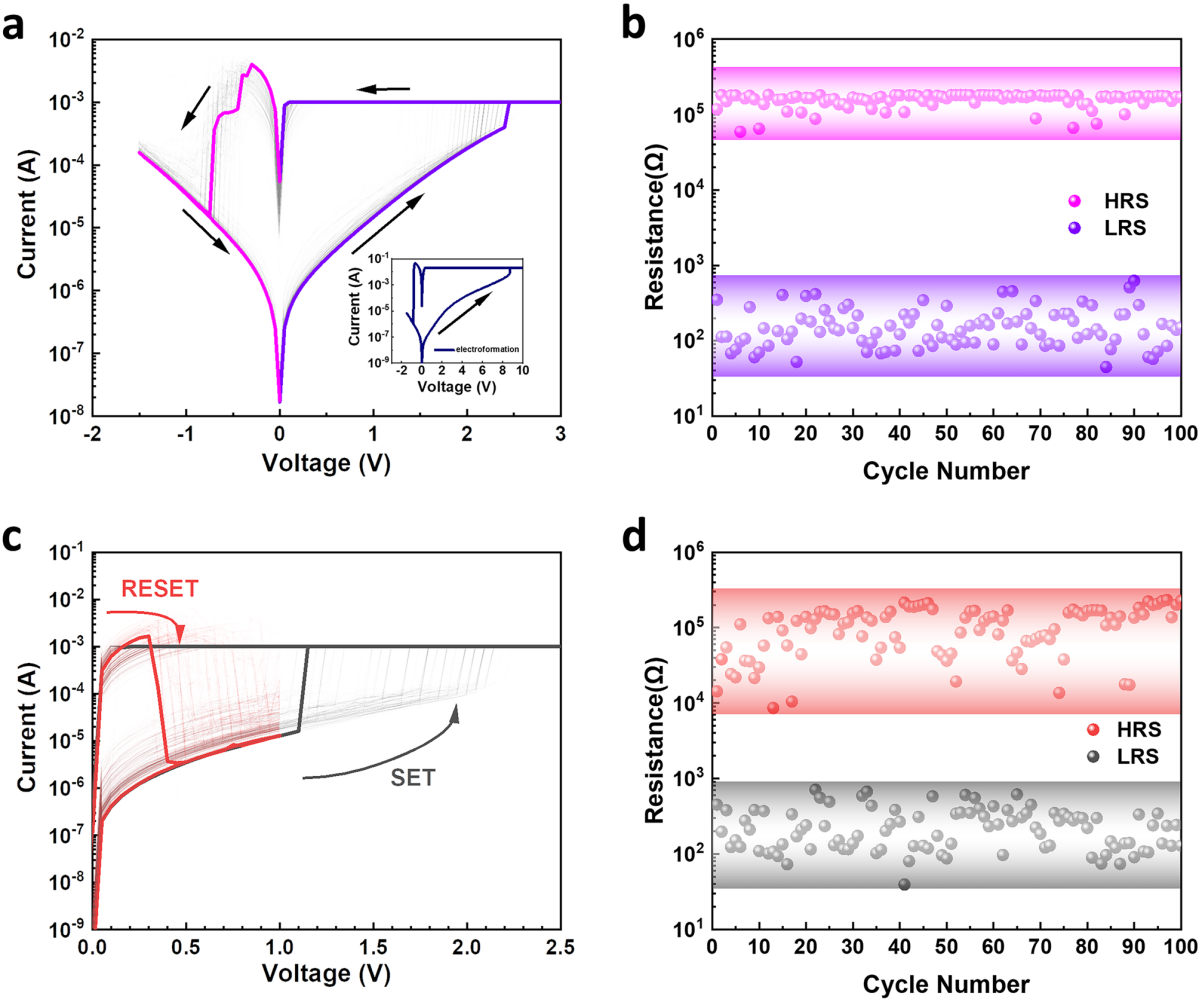
Electrical characterisation of the bilayer SiC memristor. (**a**) I–V graph of 100 bipolar DC cycles of the memristor; inset is the DC-IV curves of the electroforming cycle. (**b**) DC endurance of 100 bipolar cycles. (**c**) I–V graph 100 unipolar DC cycles. (**d**) DC endurance of 100 unipolar DC cycles.

The electrical switching properties of the memristor were first investigated by DC sweeping. Prior to the switching, an electroforming process (shown in the inset of Fig. 2a) was required to initialise the device. This was achieved by applying a large forming voltage of 8.5 V with a 10 mA current compliance (CC) to switch the device from a pristine state into a low resistance state (LRS). After switching the memristor back to the high resistance state (HRS), it can be subsequently switched on with a much lower SET voltage of *ca.* 2.5 V and a CC of 1 mA, as shown in Fig. 2a. The SET process is characterised by an abrupt increase of current to CC, after which the memristor remains at the LRS. A negative DC sweep was subsequently applied to reset the memristor back to the HRS. During the reset process, the current increased before drastically dropping back to the low level. The DC endurance of our bilayer memristor in this bipolar switching mode is presented in Fig. 2b. The device can be consistently switched for 100 cycles with an ON/OFF ratio *ca.* 3 orders of magnitude. Apart from bipolar switching, our bilayer memristor can also be switched in a unipolar manner (shown in Fig. 2c) where a positive DC sweep can also reset the device back to HRS. The DC endurance for unipolar switching mode is shown in Fig. 2d. Compared with the bipolar mode, the unipolar mode has a similar ON/OFF ratio but manifests a slightly larger resistance distribution, especially in the HRS. The co-existence of both bipolar and unipolar switching behaviours was also reported previously in SiC-based memristors^34^. In addition, such abrupt switching in both SET and RESET processes imply the switching in our memristor is filamentary dominated.

**Figure 3:**
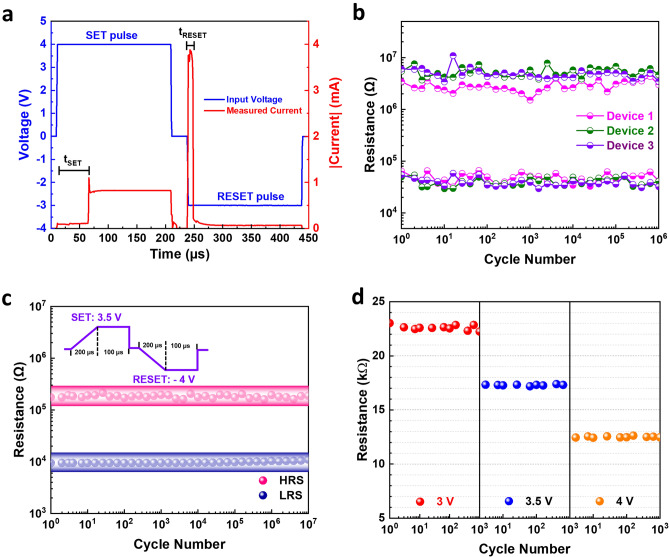
Pulse and multi-state switching of the bilayer SiC memristor (**a**) I–V–t graph showing the input SET and RESET pulses and the measured current. (**b**) Endurance of 1 million cycles of 3 different devices using pulse condition in a. (**c**) Endurance of 10 million cycles by pulses with raise time of 200 μs and duration of 100 μs. (**d**) Multiple LRS of 1000 cycles using different SET pulse voltages.

Pulsed switching was performed on our bilayer memristor. An external transistor was placed between the bottom electrode and ground to act as a current limiter in the SET process (schematic shown in Fig. S3a). The gate of the compliance circuit is floating in this measurement set-up. The inclusion of the transistor ensures an asymmetric I–V characteristic which allows the RESET current to be sufficiently high to achieve full RESET while limiting the SET current as the full I–V is shown in Fig. S3b. A SET pulse of 4 V and a RESET pulse of − 3 V were applied to switch the memristor with both pulse duration of 200 μs and rise time of 1 μs. The current measurement during pulsing is presented in Fig. 3a. It can be observed that the memristor could be switched from the HRS to the LRS in less than 70 μs whereas the RESET process registered a shorter time of *ca.* 20 μs. The associated endurance cycles of 3 memristor devices are shown in Fig. 3b where all devices can be switched repeatedly for 1 million cycles with similar and stable ON/OFF ratio. More importantly, varying the pulse parameters can control the resistance states. Figure 3c shows that by increasing the rise time of the input pulse to 200 μs and reducing the pulse width to 100 μs, resistances in both the HRS and LRS can be reduced while still maintaining similar ON/OFF ratio. More importantly, the LRS can be further fine tunned by varying the SET pulse voltage. As shown in Fig. 3d, three distinctive LRSs can be observed when the SET voltage was changed from 4 to 3.5 V and 3 V. Such capability of gradually modulate the resistance states with good endurance strongly suggest the potential application in multi-state memory and neuromorphic computing using this back-end-of-line SiC memristors.

**Figure 4:**
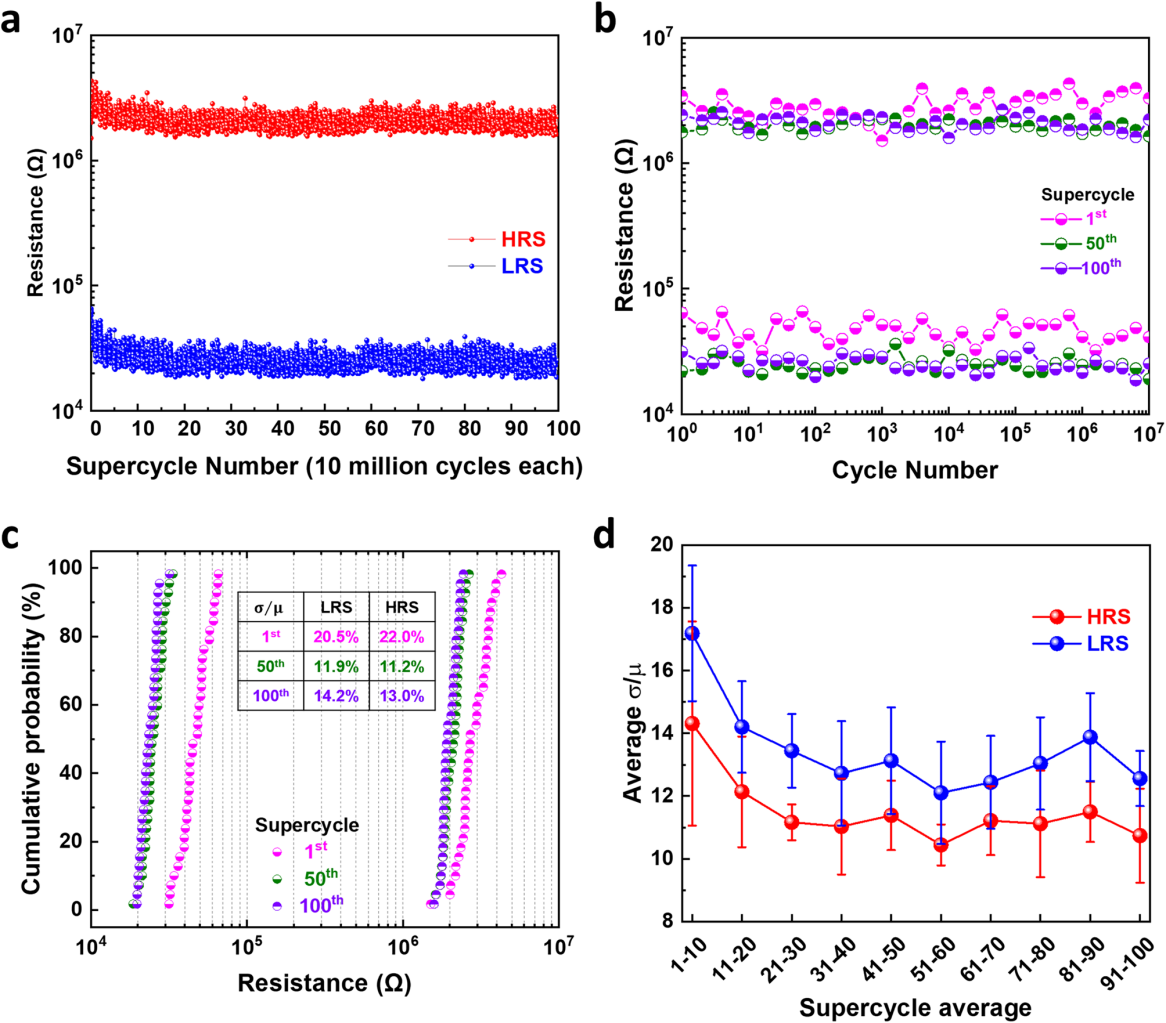
High endurance measurements of the bilayer SiC memristor. (**a**) Current-blind pulsed voltage stress (PVS) endurance measurement as a series of 100 subsequent supercycles of 10^7^ cycles each with resistance measurement of 5 times per decade (total resistance data points 3500 per state). (**b**) Endurance and (**c**) Cumulative probability of the 1st, 50th and 100th supercycle. (**d**) Average coefficient of variation of every ten supercycles.

To further explore the high endurance performance of our bilayer memristor, we tested the device by adopting a current blind pulsing approach^38^. Single pulse trains of 10 million cycles with resistance measurements five times per decade were looped over 100 times. Each 10 million loop will be considered as a supercycle. The SET and RESET Voltage remained at 4 V and − 3 V, respectively using the pulse width of 200 μs and rise/fall time of 1 μs. Figure 4a presents the HRS and LRS of 100 supercycles, demonstrating an endurance of 1 billion cycles. The endurance information within the 1st, 50th and 100th supercycles are plotted in Fig. 4b. It can be observed that both HRS and LRS in the 1st supercycle register slightly higher resistance than those of the 50th and 100th supercycles. Both states then drift and stabilise to lower values. This is confirmed by analysing the cumulative probability of both states in those supercycles in Fig. 4c. Calculation of the coefficient of variation, by taking the standard deviation and dividing it by the mean ($\sigma /\mu$), further reveals that the distribution of both HRS and LRS improve with increasing number of cycles (shown in Fig. S4). We plot the average coefficient of variation for these 100 supercycles in Fig. 4d. It can be observed that the reduction of the resistance states tends to plateau after approximately 20 supercycles. The coefficient of variations are *ca.* 11% and 12% for HRS and LRS, respectively, after 20 supercyles. This is a significant improvement to the highest reported VCM endurance, showing a variation in the HRS of two orders of magnitude^15^. This is also the first time such large number of switching cycles and thus large endurance has been reported on back-end-of-line SiC materials. No clear evidence of degradation is observed after these billion cycles. The high endurance of 1 billion cycles coupled with *ca.* 2 order of magnitude ON/OFF ratio is a superb performance for any ECM reported, let alone the memristors with strong compatibility with CMOS process.

**Figure 5:**
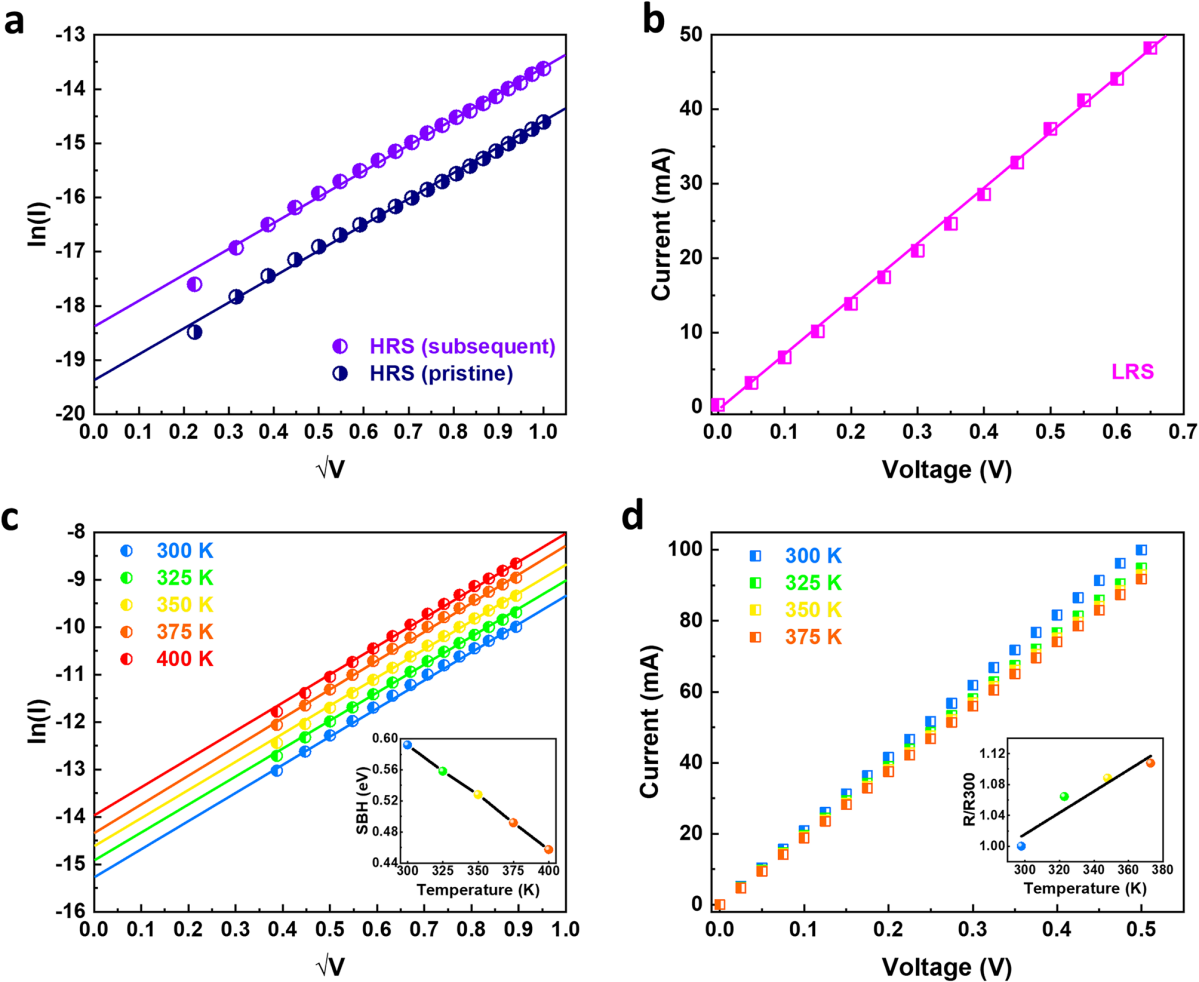
Conduction mechanism of the bilayer SiC memristor. (**a**) Schottky emission fitting for both pristine and subsequent HRS. (**b**) Ohmic conduction in the LRS. (**c**) Schottky emission fittings at varies temperatures; inset shows extracted SBHs with change in temperature (**d**) Ohmic conduction of the LRS at varying temperatures; inset shows the resistance nominated against room temperature resistance (R300) as a function of temperature.

To elucidate the switching and conduction mechanism of the memristor, we fit the bipolar *I–V* characteristics of the HRS using the Schottky emission equation:

$$I=A{A}^{*}{T}^{2}\text{exp}\left[\frac{-q{\Phi }_{B}}{kT}+\frac{q\sqrt{q/4\pi {\varepsilon }_{i}}}{kT}\sqrt{E}\right]$$

where A is the conduction area, ${A}^{*}$ is the Richardson’s constant, ${\Phi }_{B}$ is the Schottky Barrier height (SBH), $E$ is the electrical field, $q$ is the electronic charge, $k$ is the Boltzmann’s constant, ${\varepsilon }_{i}$ is the dielectric constant of the film, and $T$ is absolute temperature. Figure 5a presents the fittings for both pristine and subsequent HRS. Linear fittings in both cases imply that Schottky emission is the dominating conduction mechanism for the OFF states of the memristor. The SBHs can be extracted from the Y-axis intercepts of the linear fittings and were found to be 0.6 eV and 0.57 eV. These values are close to the SBH extracted from our previous work of single layer SiC memristor (Cu/SiC/W)^26^. This result indicates the Schottky barrier between the Cu and SiC layers still dominates the current conductions for the bilayer memristor at HRS. It is worth noting that a small SBH difference of 0.03 eV was observed between pristine and subsequent HRS, this is likely due to the leftover Cu ions in the Cu/SiC interface after the RESET process and will be discussed later. On the other hand, the current for the ON state is dominated by Ohmic conduction, as shown by the linear fitting of *I–V* curve in Fig. 5b. Unlike the single layer SiC memristor where Schottky emission still prevails at LRS at a lower SBH, the Ohmic conduction in bilayer memristor implies the formation of Cu filaments in the memristor after the SET process^26^. This assumption is also in line with the abrupt switching observed in Fig. 2. Similar current conduction mechanisms were also observed in the unipolar switching mode where the Schottky emission and Ohmic conduction dominate the OFF and ON states, respectively as shown in Fig. S5.

Temperature-dependent DC switching were further conducted to validate the switching mechanism. The bilayer memristor could be switched repeatedly even at elevated temperature up to 375 K (shown in Fig. S6). It is observed that the SET voltage decreases with increasing temperature. At higher switching temperature (e.g. > 400 K), the memristor became less stable and was hard to switch OFF. Figure 5c presents the HRS at the different temperatures in a ln(I) − √V graph. The linear fittings indicate that Schottky emission is the dominant electron movement method across the entire temperature range. The extracted SBHs was found to decrease from *ca.* 0.58 eV at room temperature to *ca.* 0.45 eV at 400 K (inset of Fig. 5c). This reduction of the barrier height with increasing temperature is likely due to the semiconductor bandgap narrowing of elevated temperatures^42^. In addition, the increase in the number of thermally excited charge carriers due to the high temperature could also serve to reduce the effective barrier height. In terms of the LRS, Ohmic conductions were observed at all temperatures with increasing temperature leading to higher resistance, as shown in Fig. 5d. By normalising the Ohmic resistances at different temperature against the room temperature resistance (*R300*), the temperature coefficient is extracted to be 1.39 × 10^−3^ K^−1^, which agrees with the temperature coefficient of resistance of Cu Nanowires^34^. This result confirms that Cu filament conduction in the LRS of our bilayer memristor.

**Figure 6:**
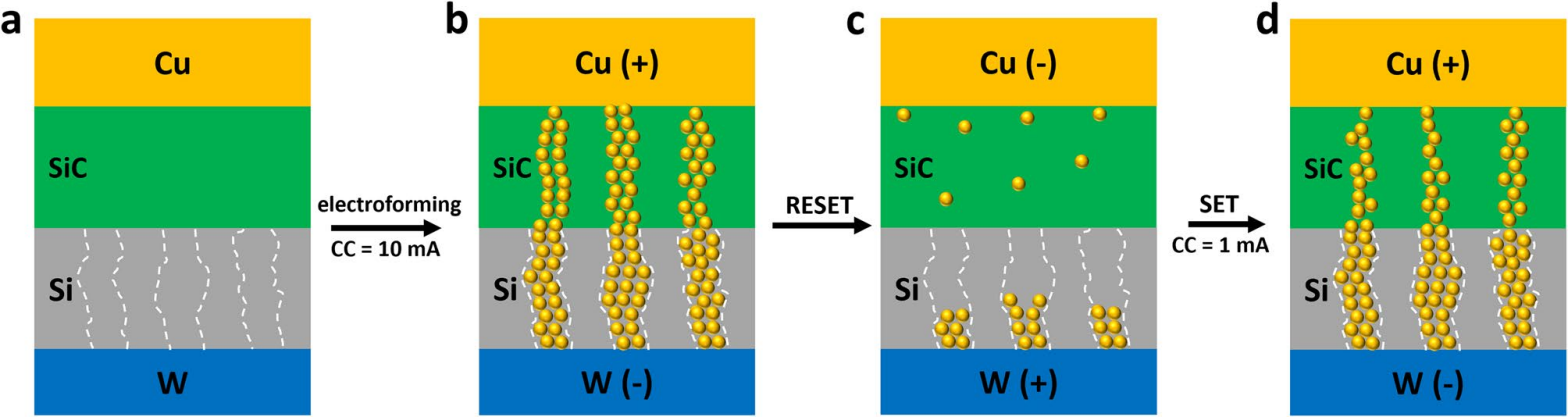
Schematic of the potential switching mechanism of the bilayer SiC memristor.

Figure 6 illustrates a full resistive switching behaviour observed in bilayer SiC/Si memristor. In the pristine state, the memristor has a high resistance which can be attributed to the high Schottky barrier at the Cu and SiC interface (Fig. 6a). Upon application of a positive bias on the Cu electrode in the electro-forming process, Cu atoms are oxidized into Cu^x+^ ions which drift through the SiC into the Si layer, and subsequently reduce to Cu atoms. In standard CBRAM devices the Cu atoms generally reduce at the inert electrode, but it has been shown that other methods of filament growth are possible depending on the ion mobility and redox rate^43,44^. It should be highlighted that the existence of vertical grain boundaries in the Si layer has been shown to serve as low-energy migration pathways for the Cu^x+^ ions^45–48^. The growth of the Cu filaments is therefore favoured within these grain boundaries in the Si layer as shown in Fig. 6b. Such defined filament growth further extends into the SiC layer and connect to the top electrode. The memristor is therefore switched into LRS with Ohmic conduction dominated by Cu filaments. It is worth noting that this forming process is critical to the switching properties of the memristors. Due to the randomness of Cu filament growth, *ca.* 50% of our devices broke down after the initial formation. However, once the device has been formed properly, it exhibits resistive switching for millions of cycles without any degradation. For the subsequent RESET process, a negative (or positive in the case of unipolar switching) bias breaks the filaments through reverse electrical field and/or Joule heating and switches the memristor back to HRS (shown in Fig. 6c). Here the HRS is again dominated by the Schottky barrier between the Cu and SiC layers. However, the rupture of filaments may leave some residual Cu ions within the Si boundaries and the SiC film after the RESET process. The latter could serve as a doping mechanism for the SiC layer which may contribute to the slight reduction of the SBH (*ca.* 0.57 eV) compared with that of the pristine state (*ca.*0.6 eV). The memristor can subsequently be switched ON again by another positive bias on the Cu electrode. The switching voltage is much smaller than the electroforming voltages due to the filament residuals in the Si grain boundaries which serve as the reduction sites for the re-growth of Cu filaments (Fig. 6d). It is worth mentioning that our previously reported single layer SiC memristor demonstrates interfacial based switching where the resistance modulation is a cause of controlled Cu doping in the SiC film during the SET and RESET process^26^. We believe that such different switching characteristic in our SiC/Si bilayer memristors is mainly attributed to the Si layer underneath where the existence of the grain boundaries provides a defined pathway that facilitates the root growth of Cu filaments across the entire bilayer. These pre-defined pathways enable the formation and rupture of filaments at localised regions which largely improves the switching stability of our SiC memristors, leading to the high endurance performance.

## Conclusion

We demonstrated a resistive memory which exploits PECVD bi-layer stack of amorphous Si and SiC as solid-state dielectric device layer, and Cu as active electrodes. The device exhibits endurance performance exceeding one billion cycles with an ON/OFF ratio of 2 orders of magnitude. The CVD deposition method of the amorphous Si/SiC layer and similarity to typical BEOL SiOC low-k interlayer dielectric makes this an advanced memory system with great promise towards CMOS integration. Multiple resistance states were successfully demonstrated by controlling the pulse conditions such as pulse time and voltage which are also important parameters for future synaptic switching applications. Further scaling and optimization of thicknesses of the layers may lead to further improvement and optimisation of variability, as well as switching currents, voltage, and speed, in order to develop towards an embedded memristor with both high-performance and neuromorphic computing potentials.

### Experimental section

#### Device fabrication

The bilayer Cu/SiC/Si/W memristor was fabricated on a SiO_2_/Si substrate. A 170 nm layer of W was firstly sputtered as the bottom electrode. The 100 nm SiC/Si bi-layer dielectric layer is deposited directly on the W layer via a PECVD process where Silane (SiH_4_) and Methane (CH_4_) were used as the reactive gases. Finally, top electrodes of the active metal Cu with approximately 100 nm thickness and variable sizes were deposited via evaporation and patterned by a photolithography and lift-off process.

### Characterisation and measurement

X-ray photoelectron spectroscopy (XPS) data were obtained using a ThermoScientific Theta Probe System with Al–Kα radiation (photon energy = 1486.6 eV). Transmission electron microscopy (TEM) specimens were prepared by mechanical polishing followed by ion milling to electron transparency using Ar + at 6 keV. A final low-energy milling step was performed at 500 eV. To minimize surface damage, the structure and morphology of the samples were analysed using a JEOL 2100 TEM equipped with LaB6 and JEOL ARM200F TEM/ scanning TEM (STEM) with a Schottky gun both operating at 200 kV. XRD patterns were collected in grazing incidence mode (θ_1_ = 1°) using a Rigaku SmartLab diffractometer (Cu Kα, λ = 1.5418 Å) with parallel X-ray beam and a Hypixdetector operated in 1D mode. The resultant memristors were characterised using both DC switching and pulsed switching. The electrical characterisation was conducted with a Keysight B1500, with the pulsed switching performed with a B1530 WGFMU pulsing module. This module has no internal current compliance feature, so an external transistor was placed between the bottom electrode and the ground. This is to act as a current limiter in the SET process, and its asymmetric behaviour allows the RESET current to be sufficiently high for full RESET.

### Supplementary Information

Supplementary Figures.

## Data Availability

The datasets generated and/or analysed during the current study are available in the University of Southampton repository at 10.5258/SOTON/D3073.
