# Supplementary material for: An ultra high-endurance memristor using back-end-of-line amorphous SiC
Source: Sci Rep. 2024 Jun 18;14:14008. doi: 10.1038/s41598-024-64499-2 (PMC11189404; doi:10.1038/s41598-024-64499-2)
Supplement: Supplementary file 1 — Supplementary Figures. [file 41598_2024_64499_MOESM1_ESM.pdf]

Supplementary Information of

An ultra high-endurance memristor using back-end-of-line amorphous SiC

Omesh Kapur[1], Dongkai Guo[1], Jamie Reynolds[1], Daniel Newbrook[1], Yisong Han[2], Richard Beanland[2], Liudi Jiang[3], C.H. Kees de Groot[1] and Ruomeng Huang[1*]
[1]School of Electronics and Computer Science, University of Southampton, Southampton, SO17 1BJ, United Kingdom
[2]Department of Physics, University of Warwick, Coventry, CV4 7AL, United Kingdom
[3]School of Engineering, University of Southampton, Southampton SO17 1BJ, United Kingdom

*Correspondence to r.huang@soton.ac.uk

Figure S1 shows the X-ray Diffraction analysis on our SiC/Si bilayer structure. While no SiC peaks were observed as the film was amorphous as-deposited, several peaks associated with polycrystalline silicon can be identified. These peaks at 28.1°, 47.4° and 54.6° correspond to the (111), (220) and (311) planes of Si. A few other peaks from the underneath W bottom electrode are also presented. The polycrystalline nature of the Si film further supports the existence of grain boundaries in the layer.

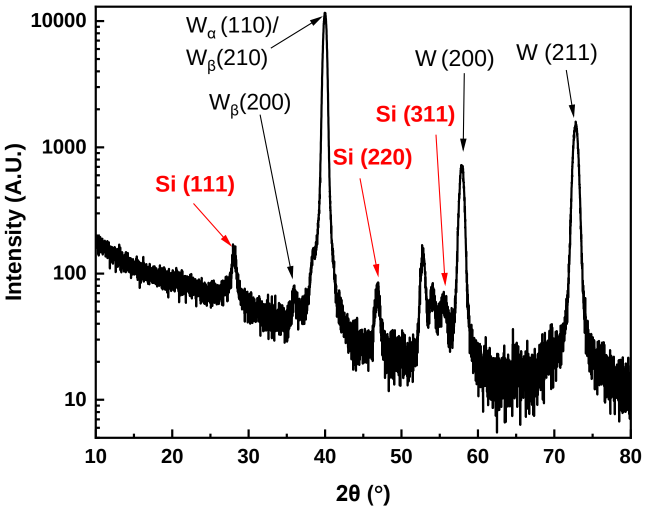

Figure S1 X-Ray Diffraction of the SiC/Si bilayer structure films. The peaks around 28.1°, 47.4° and 54.6° correspond to the (111), (220) and (311) planes of Si. The bottom W electrode also displays significant peaks in the results.

Figure S2a presents the XPS depth profile of the SiC/Si bilayer structure. The SiC and Si layers can be clearly distinguished. The Si:C ratio in the SiC layer was found to be 7:3 which is in good agreement with the EDX result. The survey spectra for both SiC and Si layers are shown in Figure S2b where no impurities can be observed. The absence of C peak in the Si layer is clearly visible.

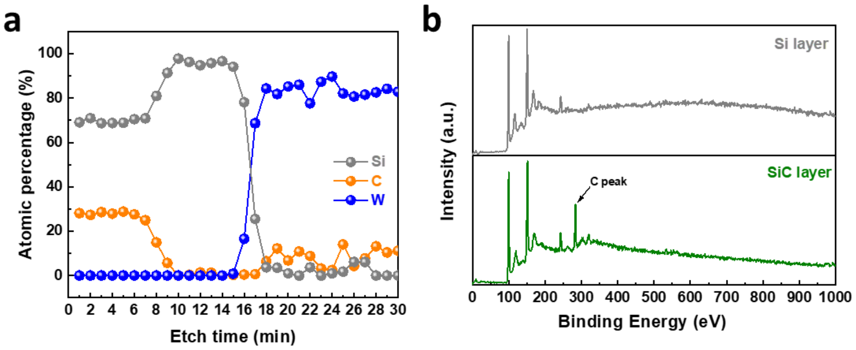

Figure S2 (a) XPS depth profile of the SiC/Si bilayer structure on top of a W layer. (b) XPS survey spectra for both SiC and Si layers.

Figure S3a illustrates the circuit's schematic in which a transistor is connected between the bottom electrode of the memristor and the ground to serve as the current limiter. The *I-V* characteristic of the transistor without the application of gate voltage is shown in Figure S3b. It can be observed that the transistor exhibits the required 1 mA current at positive bias for SET process, and greater than 1 mA current at negative bias for RESET process. The *I-V* curve for transistor-memristor is also plotted in the same figure where similar bipolar switching can be observed without the need of a compliance current. It is worth mentioning that the transistor reduces the ON/OFF ratio to two orders of magnitude due to the resistance across both devices in the LRS. The device can still switch consistently as with the previously measured DC characterisation. This current limiting setup provides the ability to performance extremely long endurance testing by pulsing.

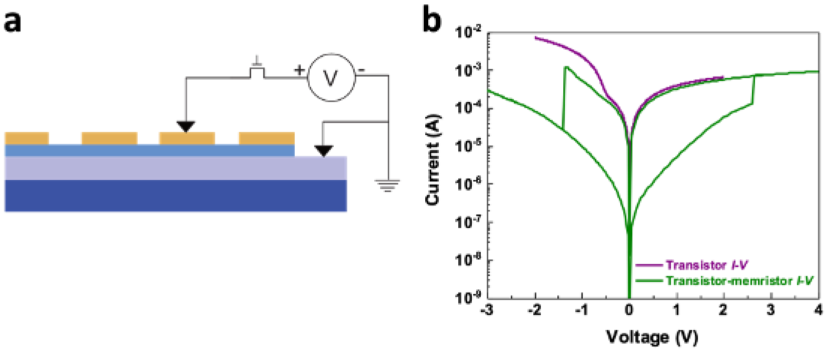

Figure S3 (a) Schematics of a compliance current circuit in series with a transistor connected in series of our bilayer SiC memristor. (b) I-V characteristic of the transistor and transistor-memristor circuit.

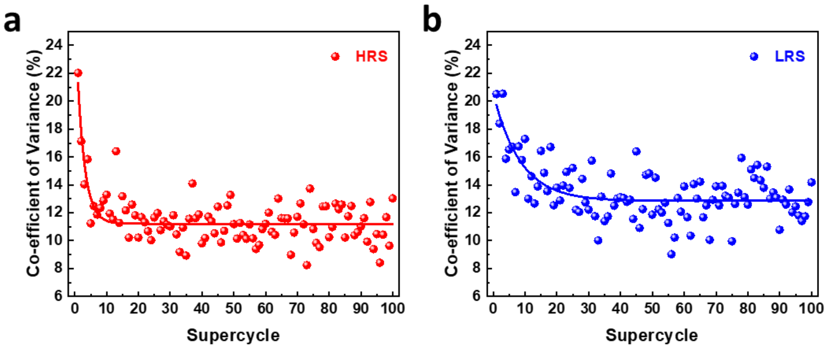

Figure S4 Average coefficient of variations of each supercycle for (a) HRS and (b) LRS of the bilayer SiC memristor. The red lines serve as guides of eyes.

When analysing the Schottky emissions between bipolar and unipolar switching modes, the HRS shows a near-identical response, with both having a barrier height of *ca.* 0.57eV as shown in Figure S5a. This is expected as the barrier height should remain the same between the two modes. The Ohmic response at LRS is also very similar, with only a slight deviation (shown in Figure S5b). This is possibly due to a slight difference in the LRS value caused by a stronger or weaker connection to the bottom electrode.

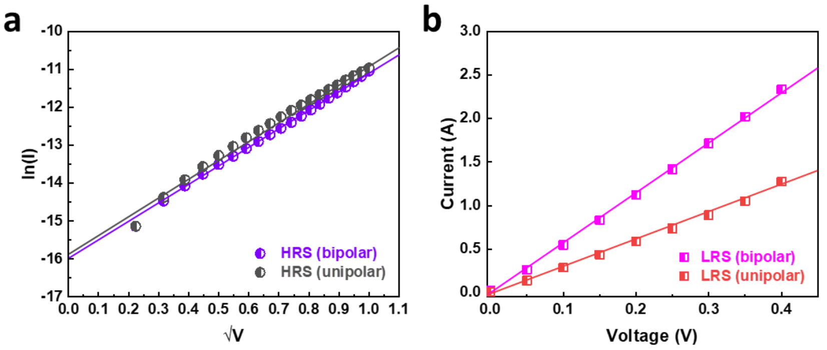

Figure S5 (a) Schottky emission fitting for both bipolar and unipolar switching modes at HRS. (b) Ohmic conduction fitting for both bipolar and unipolar switching modes at LRS.

Figure S6a presents the DC bipolar switching of our SiC bilayer memristor at different temperatures from 300 K to 400 K. Above this temperature, the device becomes unstable and must be set numerous times to retain its bit state. At 400 K, the device cannot entirely switch to the LRS. Figure S6b plots the DC endurance of memristors with different device dimensions. It can be observed that the LRS demonstrates an area independent behaviour, a typical characteristic of filamentary switching. On the other hand, the HRS is dependent on the device dimension.

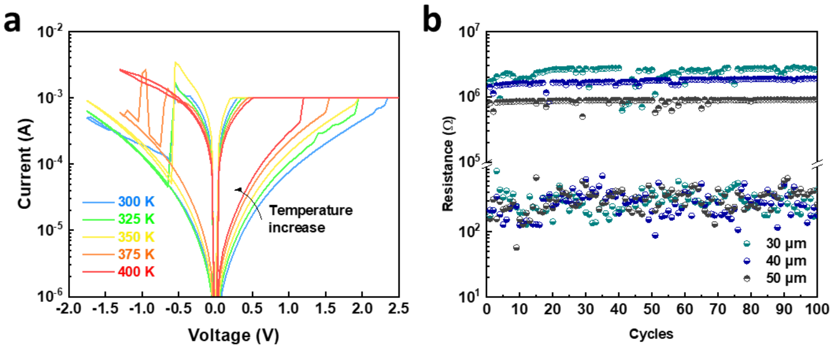

Figure S6 (a) Temperature dependant switching between 300 K and 400 K. (b) DC endurance of the SiC bilayer memristor with different device dimensions.
